# Autoinflammatory genes and susceptibility to psoriatic juvenile idiopathic arthritis

**DOI:** 10.1002/art.23604

**Published:** 2008-07

**Authors:** T G Day, A V Ramanan, A Hinks, R Lamb, J Packham, C Wise, M Punaro, R P Donn

**Affiliations:** 1 University of ManchesterManchester, UK; 2 Bristol Royal Hospital for ChildrenBristol, UK and Royal National Hospital for Rheumatic DiseasesBath, UK; 3 Haywood Hospital, Stoke-on-TrentUK; 4 Texas Scottish Rite Hospital for ChildrenDallas

## Abstract

**Objective:**

To investigate the association of *NLRP3, NOD2, MEFV,* and *PSTPIP1*, genes that cause 4 of the autoinflammatory hereditary periodic fever syndromes (HPFS), with juvenile idiopathic arthritis (JIA).

**Methods:**

Fifty-one single-nucleotide polymorphisms (SNPs) across the 4 loci were investigated using MassArray genotyping in 950 Caucasian patients with JIA living in the UK and 728 ethnically matched healthy controls.

**Results:**

Prior to Bonferroni correction for multiple testing, significant genotype associations between 6 SNPs in *MEFV* and JIA were observed and, in subgroup analysis, associations between 12 SNPs across all 4 loci and the subgroup of patients with psoriatic JIA were found. After Bonferroni correction for multiple testing, 2 genotype associations remained significant in the subgroup of patients with psoriatic JIA (*MEFV* SNP rs224204 [corrected *P* = 0.025] and *NLRP3* SNP rs3806265 [corrected *P* = 0.04]).

**Conclusion:**

These findings support the use of monogenic loci as candidates for investigating the genetic component of complex disease and provide preliminary evidence of association between SNPs in autoinflammatory genes and psoriatic JIA. Our findings raise the interesting possibility of a shared disease mechanism between the HPFS and psoriatic JIA, potentially involving abnormal production of interleukin-1β.

Juvenile idiopathic arthritis (JIA) is the most common rheumatic disease of childhood. It is characterized by chronic inflammation of one or more synovial joints, with onset before the age of 16 years and duration of >6 weeks (1). One approach to the selection of JIA candidate genes is to use genes responsible for monogenic syndromes that have an overlap of phenotype with JIA. This has been successful previously, with the genes *WISP3* and *SLC26A2* shown to confer susceptibility to specific subgroups of JIA (2,3). Continuing this strategy, we investigated the following genes, which are responsible for 4 of the autoinflammatory hereditary periodic fever syndromes (HPFS): *NLRP3* (*CIAS1/*cryopyrin*/NALP3/PYPAF1*), responsible for cryopyrin-associated periodic syndrome; *NOD2* (*CARD15)*, responsible for Blau syndrome; *MEFV* (marenostrin*/*pyrin), responsible for familial Mediterranean fever; and *PSTPIP1* (*CD2BP1/PSTPIP*), responsible for pyogenic arthritis, pyoderma gangrenosum, and acne (PAPA) syndrome (4). The HPFS are a group of monogenic autoinflammatory syndromes, unified by a central phenotype of recurrent inflammation, often manifested as unexplained fever, coupled with other signs of systemic infection. Arthralgia or arthritis is often present (4).

Certain characteristics are common to both HPFS and JIA, especially systemic-onset JIA. These include shared clinical features related to systemic inflammation, and a shared response to treatment with interleukin-1 receptor antagonist (IL-1Ra) (anakinra), suggesting a similar pathogenesis. In addition, polymorphisms in *NALP1*, a gene related to the HPFS gene *NLRP3*, were recently shown to confer susceptibility to vitiligo-associated autoimmune disease (5). This is relevant because the pathogenesis of JIA includes a substantial autoimmune element. It was our hypothesis that while severe mutations in the above genes will cause the respective monogenic syndrome, milder genetic changes (e.g., single-nucleotide polymorphisms [SNPs]) may give rise to a more subtly altered protein, and hence contribute to the oligogenetic basis of JIA.

## PATIENTS AND METHODS

### Patients and controls

Blood samples were obtained after subjects gave informed consent. Ethics approval was obtained from the Multi-centre Research Ethics Committee (MREC 99/8/84) and the University of Manchester Committee on the Ethics of Research on Human Beings (8/92/[i] [b]). Patients were classified according to the International League of Associations for Rheumatology criteria (1).

DNA samples obtained from 950 patients with JIA were available for genotyping of *NLRP3, NOD2,* and *MEFV*. Of the 950 patients, 175 had systemic-onset JIA, 219 had persistent oligoarticular JIA, 152 had extended oligoarticular JIA, 185 had rheumatoid factor (RF)–negative polyarticular JIA, 65 had RF-positive polyarticular JIA, 79 had enthesitis-related JIA, 67 had psoriatic JIA, and 8 had unclassifiable JIA. Samples obtained from 831 patients with JIA were available for genotyping *PSTPIP1.* Of these patients, 133 had systemic-onset JIA, 215 had persistent oligoarticular JIA, 127 had extended oligoarticular JIA, 168 had RF-negative polyarticular JIA, 48 had RF-positive polyarticular JIA, 58 had enthesitis-related JIA, 57 had psoriatic JIA, and 25 had unclassifiable JIA. In addition, 728 unrelated, ethnically matched healthy controls were genotyped for *NLRP3,* *NOD2,* and *MEFV*, and 628 for *PSTPIP1*. Controls were blood donors from the Oxford region, subjects selected from the registries of general practitioners in Norfolk, or members of a cohort of population-based controls collected in the northwest region of England.

### SNP selection

Pairwise haplotype-tagging SNPs were selected for each gene using HapMap (www.hapmap.org) and the tagger function in Haploview, version 3.32 software. This panel was enriched with SNPs in functional domains (www.ncbi.nih.gov/SNP; http://fmf.igh.cnrs.fr/ISSAID/infevers).

### Genotyping

Fifty-one SNPs (21 in *NLRP3,* 13 in *NOD2*, 11 in *MEFV*, and 6 in *PSTPIP1)* were genotyped by MassArray DNA analysis. Amplifications were conducted according to the recommendations of the manufacturer (Sequenom, San Diego, CA).

### Statistical analysis

Prior to analysis, data for DNA samples that failed to genotype in ≥50% of the SNPs assayed and data for SNPs that failed to genotype in ≥10% of the samples were removed. Hardy-Weinberg equilibrium was determined separately for patients and controls using Stata, version 9 (StataCorp, College Station, TX). SNPs were excluded from further analysis if deviation from Hardy-Weinberg equilibrium (*P* ≤ 0.05) was observed in the controls.

### Single-point analysis

Remaining SNPs were analyzed by the chi-square test (or Fisher's exact test when n ≤ 5), using Stata, version 9. Genotype and allele associations, models of dominant and recessive traits, and trend tests were investigated. Exploratory uncorrected *P* (*P*_uncorr_) values are presented in Tables 1 and 2 and Supplementary Table 1 (available on the *Arthritis & Rheumatism* Web site at http://www.mrw.interscience.wiley.com/suppmat/0004-3591/suppmat/). *P*_uncorr_ values of less than or equal to 0.05 were considered to be preliminary evidence of association. Initially, the entire group of JIA patients was compared with controls, and then each subgroup of JIA patients was compared with controls.

**Table 1:** Genotype frequencies of *MEFV* SNPs in JIA patients and controls*

	JIA patients			Controls			
SNP	Sample size	*P*†	Genotype frequency	Sample size	*P*†	Genotype frequency	*P*‡
rs2741919	782	0.43		723	0.07		0.042
TT			239 (30.6)			263 (36.4)	
TC			397 (50.8)			326 (45.1)	
CC			146 (18.7)			134 (18.5)	
rs224204	783	0.06		721	0.06		0.005
TT			178 (22.7)			213 (29.5)	
TC			418 (53.4)			334 (46.3)	
CC			187 (23.9)			174 (24.1)	
rs224213	770	0.71		722	0.16		0.143
TT			248 (32.2)			267 (37.0)	
TC			383 (49.7)			329 (45.6)	
CC			139 (18.1)			126 (17.5)	
rs224215	714	0.12		699	0.43		0.028
TT			280 (39.2)			257 (36.8)	
TC			350 (49.0)			325 (46.5)	
CC			84 (11.8)			117 (16.7)	
rs224217	772	0.34		719	0.10		0.008
TT			227 (29.4)			195 (27.1)	
TC			396 (51.3)			337 (46.9)	
CC			149 (19.3)			187 (26.0)	
rs224223	709	0.88		691	0.20		0.043
CC			207 (29.2)			181 (26.2)	
CA			355 (50.1)			328 (47.5)	
AA			147 (20.7)			182 (26.3)	
rs224225	777	0.52		722	0.10		0.014
TT			230 (29.6)			195 (27.0)	
TC			394 (50.7)			339 (47.0)	
CC			153 (19.7)			188 (26.0)	

* Except where indicated otherwise, values are the number (%). SNPs = single-nucleotide polymorphisms; JIA = juvenile idiopathic arthritis.

† For Hardy-Weinberg equilibrium.

‡ Uncorrected *P* value for genotype association.

**Table 2:** Genotype association results obtained using genotype data from the present study only and from both the present study and the Wellcome Trust Case Control Consortium to increase study power*

			*P* for all JIA†		*P* for psoriatic JIA‡	
Gene/SNP	No. of controls (present study only)	Total no. of controls	Data from present study only	All data	Data from present study only	All data
*NLRP3*/rs10754555	696	3,627	0.666	0.794	0.133	0.205
*NOD2*/rs1861759	715	3,648	0.309	0.05	0.031	0.009
*NOD2*/rs751271	724	3,523	0.239	0.259	0.053	0.075
*NOD2*/rs313499	723	3,634	0.212	0.023	0.084	0.069
*PSTPIP1*/rs2254441§	551	3,446	0.539	0.109	0.016	0.006

* Genotype data (n = 3,000) from the Wellcome Trust Case Control Consortium were merged with the control cohort data from the present study, after comparing the 2 cohorts using the chi-square test to ensure that they were statistically similar.

† Uncorrected *P* value for all patients with juvenile idiopathic arthritis (JIA) versus controls. *P* values of less than or equal to 0.05 were considered significant.

‡ Uncorrected *P* value for patients with psoriatic JIA versus controls. *P* values of less than or equal to 0.05 were considered significant.

§ A perfect proxy single-nucleotide polymorphism (SNP) for rs2254441 (rs1022197; r^2^ = 1) was found in the Wellcome Trust Case Control Consortium data.

### Wellcome Trust Case Control Consortium data

Genotype data from a study of 500,000 SNPs across the genome have recently been made publicly available (6). For SNPs (or perfect proxies, i.e., SNPs correlated with a correlation coefficient of 1 [r^2^ = 1]) that were included in both the Wellcome Trust Case Control Consortium study and our study, genotype frequencies from the control cohort of ∼3,000 individuals were compared with our control cohort using the chi-square test. If statistically similar, the 2 cohorts were merged and genotype frequencies were compared with the JIA patients in the present study, both as a whole and by individual subgroup.

### Correction for multiple testing

Bonferroni correction was performed, based on the 36 SNPs presented that passed quality control criteria.

### Multipoint analysis

Linkage disequilibrium was calculated using Haploview, version 3.32. Evaluation of association of haplotypes was carried out using a 2-marker sliding window approach, implemented using Plink, version 0.99r. Since *MEFV* and *PSTPIP1* protein products are known to interact (7), epistasis analysis, or analysis of evidence of gene–gene interaction, was conducted using Plink, version 0.99r.

### Power calculations

Power calculations for the study were carried out using Quanto software (http://hydro.usc.edu/GxE). Based on the smallest cohort sizes (831 patients and 628 controls), the study had 84% power to detect an odds ratio (OR) of ≥1.4 at the 5% significance level. Using the Wellcome Trust Case Control Consortium control genotype data for ∼3,000 additional controls (as described below) and our entire JIA cohort (n = 950), the study had 81% power to detect an OR of ≥1.3.

## RESULTS

Fifty-one SNPs were genotyped across the 4 loci. Fifteen SNPs were excluded from further analysis, due to either complete genotyping failure (rs10925015, rs7525979, rs3135500), having a minor allele frequency of <1% (rs2066844, despite the published HapMap minor allele frequency of 0.22), deviating from Hardy-Weinberg equilibrium in controls (rs4925659, rs10925027, rs2066843, rs224234, rs2741918, rs224207, rs224208, rs3935339), or falling below the required 90% SNP genotyping success rate (rs4612666, rs10159239, rs10754558).

Genotype frequencies of the remaining SNPs were compared between JIA patients and controls. *P*_uncorr_ values showed preliminary evidence of association between JIA and 6 SNPs, all of which were in the *MEFV* gene (Table 1). However, following correction for multiple testing, no significant associations were observed.

Similarly, prior to correction for multiple testing, significant associations between 12 SNPs across all 4 loci and psoriatic JIA were observed (Supplementary Table 1). In addition, an association between SNP rs4078354 in *PSTPIP1* and systemic-onset JIA was observed (*P*_uncorr_ = 0.026). Associations were also observed between persistent oligoarticular JIA and rs224225 in *MEFV* (*P*_uncorr_ = 0.022) and rs224217 in *MEFV* (*P*_uncorr_ = 0.031), between extended oligoarticular JIA and rs224217 in *MEFV* (*P*_uncorr_ = 0.047), and between RF-negative polyarticular JIA and rs224215 in *MEFV* (*P*_uncorr_ = 0.009).

After applying the stringent Bonferroni correction for multiple testing, 2 SNPs, rs224204 in *MEFV* and rs3806265 in *NLRP3*, remained significantly associated by genotype. Both were associated with psoriatic JIA (corrected *P* = 0.025 for rs224204 and corrected *P* = 0.04 for rs3806265).

Haplotype association analysis was carried out for SNPs in all loci, but no haplotype association of an order of magnitude greater than the single-point association was observed. Also, no significant epistatic interaction between *MEFV* and *PSTPIP1* was found.

Four SNPs from our study were also included in the Wellcome Trust Case Control Consortium study, and 1 SNP was covered by a perfect proxy SNP (r^2^ = 1), with genotype frequencies that were not statistically significantly different between the 2 control cohorts (Table 2). Control genotype data were therefore merged and compared with the JIA patient data, both as a whole and by individual subgroup. Two SNPs (rs1861759 and rs313499), both in *NOD2*, which were previously not significantly associated with JIA, were then found to be significantly associated with JIA overall (*P*_uncorr_ *=* 0.05 and *P*_uncorr_ = 0.023, respectively). For 1 of these SNPs (rs1861759), and for the SNP covered by the perfect proxy (rs2254441 in *PSTPIP1*), the previously observed association with psoriatic JIA was maintained with an increased significance level (*P*_uncorr_ *=* 0.009 and *P*_uncorr_ *=* 0.006, respectively) (Table 2).

## DISCUSSION

*NLRP3*, *NOD2*, *MEFV,* and *PSTPIP1* are the genes responsible for 4 of the syndromes collectively termed hereditary periodic fever syndromes (HPFS) (4). These are autoinflammatory, monogenic syndromes, characterized by recurrent and seemingly unprovoked episodes of inflammation and fever (8). Because of the overlap between these conditions and JIA, specifically systemic-onset JIA, we hypothesized that SNPs within these genes may confer susceptibility to JIA. However, rather than finding associations with systemic-onset JIA, we unexpectedly observed associations with psoriatic JIA.

We increased the size of our control cohort by using genotype data from the Wellcome Trust Case Control Consortium (6). By increasing the power of the study in this way, we uncovered additional associations between SNPs in *NOD2* and JIA, although these were not significant once Bonferroni correction for multiple testing was applied.

The 4 genes that were studied all encode for proteins that are fundamental components of the innate immune system (4). These proteins are thought to contribute to an intracellular pathogen-sensing mechanism, the output of which is increased production and secretion of IL-1β, via activation of caspase 1 (4).

The IL-1 family has been shown to play an important role in the pathogenesis of psoriasis. Overexpression of IL-1α in basal epidermis in transgenic mice initiates a psoriasis-like cutaneous inflammation (9). Moreover, deletion of the IL-1Ra gene in transgenic mice has been shown to induce psoriasiform cutaneous inflammation (10).

It is possible that polymorphisms in the genes investigated in this study may have downstream effects on IL-1β production and processing, and subsequently affect susceptibility to psoriatic JIA. IL-1Ra (anakinra) is used as an effective treatment for both HPFS and systemic-onset JIA. If our data are replicated in additional studies, it is possible that treatment with IL-1β antagonists could be extended to psoriatic JIA, as a novel and potentially effective treatment.

Two of the genes used in this study have previously been shown to be associated with complex autoimmune diseases other than JIA. *NOD2* confers susceptibility to Crohn's disease (11). *NOD2* controls both innate and adaptive immune responses, through the regulation of cytokines, chemokines, and antimicrobial peptide production. The mechanism by which genetic variation in *NOD2* confers disease susceptibility has yet to be fully elucidated (12). It has been suggested that *MEFV* is a predisposing factor for Behçet's disease (13). Furthermore, mutations within *MEFV* have previously been described in both juvenile and adult inflammatory arthritis (14,15). Also, *NALP1*, a homolog of *NLRP3*, has recently been shown to be associated with vitiligo-associated multiple autoimmune disease (5).

Although JIA is the most common chronic rheumatic disease of childhood, it is a rare condition, affecting only 1 in 10,000 children under the age of 16 years. While our total JIA cohort is quite large, numbers in any one subgroup are relatively small. Additional support for these findings is required, in the form of replication cohorts and functional studies.

The SNPs rs4078354 and rs8030698 within *PSTPIP1* have been studied in a discovery cohort of psoriatic JIA patients from Texas. No significant trends were seen (Wise C: personal communication). However, the sample size in that cohort was too small to adequately test replication, and further study in a larger cohort is still required in order to confirm or refute our findings.

In summary, we studied a large group of Caucasian JIA patients living in the UK and ethnically matched healthy controls and found preliminary evidence of association of *NLRP3*, *NOD2*, *MEFV*, and *PSTPIP1* with psoriatic JIA. These findings support the value of extrapolating from monogenic syndromes to identify candidate susceptibility genes for complex disease. Further studies, in different populations of psoriatic JIA patients, should be performed to replicate these findings.

## AUTHOR CONTRIBUTIONS

Dr. Donn had full access to all of the data in the study and takes responsibility for the integrity of the data and the accuracy of the data analysis.

**Study design.** Day, Ramanan, Donn.

**Acquisition of data.** Day, Hinks, Lamb, Packham, Wise, Punaro.

**Analysis and interpretation of data.** Day, Ramanan, Lamb, Wise, Donn.

**Manuscript preparation.** Day, Ramanan, Packham, Punaro, Donn.

**Statistical analysis.** Day, Hinks, Lamb, Wise, Donn.
