# Supplementary material for: Autoinflammatory genes and susceptibility to psoriatic juvenile idiopathic arthritis
Source: Arthritis Rheum. 2008 Jul;58(7):2142–6. doi: 10.1002/art.23604 (PMC2688675; doi:10.1002/art.23604)
Supplement: Supplementary file 1 [file art0058-2142-SD1.doc]

**Supplementary Table 1.** Genotype frequencies in JIA subgroups versus controls for SNPs within NLRP3, NOD2, MEFV and PSTPIP1 showing preliminary evidence of association*

| SNP,  (gene),  genotype | Systemic-onset JIA | | Persistent oligoarticular JIA | | Extended oligoarticular JIA | | RF-negative polyarticular JIA | | | RF-positive polyarticular JIA | | | | Enthesitis-related JIA | | | | Psoriatic JIA | | | Controls  n= (%) | |  |
| --- | --- | --- | --- | --- | --- | --- | --- | --- | --- | --- | --- | --- | --- | --- | --- | --- | --- | --- | --- | --- | --- | --- | --- |
|  | n= (%) | Puncorr | n= (%) | P uncorr | n= (%) | P uncorr | n= (%) | P uncorr | | | n=(%) | Puncorr | | n= (%) | | P uncorr | | n= (%) | P uncorr | |  |  |  |
| rs3806265 (*NLRP3)* |  |  |  |  |  |  |  |  | | |  |  | |  | |  | |  |  | |  | |  |
| TT | 65 (46.4) | 0.749 | 79 (43.4) | 0.978 | 49 (36.6) | 0.322 | 81 (50.0) | 0.294 | | | 28 (50.0) | | 0.643 | | 24 (37.5) | | 0.643 | 14 (28.6) | | ***0.001†*** | | 315 (43.6) | |
| TC | 60 (42.9) |  | 81 (44.5) |  | 66 (49.3) |  | 65 (40.1) |  | | | 22 (39.3) | |  | | 31 (48.4) | |  | 34 (69.4) | |  | | 317 (43.8) | |
| CC | 15 (10.7) |  | 22 (12.1) |  | 19 (14.2) |  | 16 (9.9) |  | | | 6 (10.7) | |  | | 9 (14.1) | |  | 1 (2.0) | |  | | 91 (12.6) | |
| rs12564791  (*NLRP3)* |  |  |  |  |  |  |  |  | | |  | |  | |  | |  |  | |  | |  | |
| CC | 111 (82.8) | 0.095 | 125 (72.3) | 0.549 | 103 (79.8) | 0.384 | 118 (74.7) | 0.947 | | | 37 (67.3) | | 0.144 | | 47 (81.0) | | 0.306 | 26 (55.3) | | ***0.011†*** | | 527 (74.1) | |
| CT | 20 (14.9 |  | 45 (26.0) |  | 23 (17.8) |  | 35 (22.2) |  | | | 18 (32.7) | |  | | 11 (19.0) | |  | 20 (42.6) | |  | | 164 (23.1) | |
| TT | 3 (2.2) |  | 3 (1.7) |  | 3 (2.3) |  | 5 (3.2) |  | | | 0 (0.0) | |  | | 0 (0.0) | |  | 1 (2.1) | |  | | 20 (2.8) | |
| rs2067085  (*NOD2)* |  |  |  |  |  |  |  |  | | |  | |  | |  | |  |  | |  | |  | |
| CC | 52 (40.3) | 0.347 | 57 (35.2) | 0.327 | 48 (39.3) | 0.429 | 46 (34.1) | 0.882 | | | 17 (31.5) | | 0.882 | | 20 (38.5) | | 0.778 | 9 (19.6) | | ***0.008†*** | | 235 (33.9) | |
| CG | 57 (44.2) |  | 87 (53.7) |  | 54 (44.3) |  | 70 (51.9) |  | | | 25 (46.3) | |  | | 25 (48.1) | |  | 34 (73.9) | |  | | 349 (50.4) | |
| GG | 20 (15.5) |  | 18 (11.1) |  | 20 (16.4) |  | 19 (14.1) |  | | | 12 (22.2) | |  | | 7 (13.5) | |  | 3 (6.5) | |  | | 109 (15.7) | |
| rs1861759  (*NOD2)* |  |  |  |  |  |  |  |  | | |  | |  | |  | |  |  | |  | |  | |
| AA | 53 (38.7) | 0.62 | 65 (36.7) | 0.112 | 53 (40.8) | 0.356 | 56 (35.4) | 0.483 | | | 16 (29.6) | | 0.483 | | 22 (36.1) | | 0.853 | 12 (23.5) | | ***0.031†*** | | 246 (34.4) | |
| AC | 63 (46.0) |  | 95 (53.7) |  | 57 (43.8) |  | 83 (52.5) |  | | | 26 (48.1) | |  | | 31 (50.8) | |  | 35 (68.6) | |  | | 356 (49.8) | |
| CC | 21 (15.3) |  | 17 (9.6) |  | 20 (15.4) |  | 19 (12.0) |  | | | 12 (22.2) | |  | | 8 (13.1) | |  | 4 (7.8) | |  | | 113 (15.8) | |
| rs224204  *(MEFV)* |  |  |  |  |  |  |  |  | | |  | |  | |  | |  |  | |  | |  | |
| TT | 36 (25.9) | 0.68 | 37 (21.1) | 0.084 | 33 (25.0) | 0.483 | 43 (26.2) | 0.297 | | | 9 (16.7) | | 0.07 | | 13 (20.3) | | 0.291 | 6 (12.0) | | ***0.001†*** | | 213 (29.5) | |
| TC | 67 (48.2) |  | 90 (51.4) |  | 68 (51.5) |  | 87 (53.0) |  | | | 33 (61.1) | |  | | 33 (51.6) | |  | 37 (74.0) | |  | | 334 (46.3) | |
| CC | 36 (25.9) |  | 48 (27.4) |  | 31 (23.5) |  | 34 (20.7) |  | | | 12 (22.2) | |  | | 18 (28.1) | |  | 7 (14.0) | |  | | 174 (24.1) | |
| rs224215  *(MEFV)* |  |  |  |  |  |  |  |  | | |  | |  | |  | |  |  | |  | |  | |
| TT | 54 (40.9) | 0.488 | 56 (34.1) | 0.719 | 45 (35.7) | 0.13 | 66 (47.5) | ***0.009†*** | | | 24 (43.6) | | 0.539 | | 21 (38.9) | | 0.35 | 12 (30.0) | | 0.394 | | 257 (36.8) | |
| TC | 61 (46.2) |  | 82 (50.0) |  | 68 (54.0) |  | 62 (44.6) |  | | | 24 (43.6) | |  | | 28 (51.9) | |  | 23 (57.5) | |  | | 325 (46.5) | |
| CC | 17 (12.9) |  | 26 (15.9) |  | 13 (10.3) |  | 11 (7.9) |  | | | 7 (12.7) | |  | | 5 (9.3) | |  | 5 (12.5) | |  | | 117 (16.7) | |
| rs224217  *(MEFV)* |  |  |  |  |  |  |  |  | | |  | |  | |  | |  |  | |  | |  | |
| TT | 43 (31.9) | 0.27 | 47 (26.4) | ***0.031†*** | 47 (35.3) | ***0.047†*** | 52 (32.9) | 0.3 | | | 14 (25.0) | | 0.605 | | 15 (23.4) | | 0.486 | 6 (14.0) | | ***0.006†*** | | 195 (27.1) | |
| TC | 65 (48.1) |  | 100 (56.2) |  | 63 (47.4) |  | 71 (44.9) |  | | | 30 (53.6) | |  | | 35 (54.7) | |  | 31 (72.1) | |  | | 337 (46.9) | |
| CC | 27 (20.0) |  | 31 (17.4) |  | 23 (17.3) |  | 35 (22.2) |  | | | 12 (21.4) | |  | | 14 (21.9) | |  | 6 (14.0) | |  | | 187 (26) | |
| rs224223  *(MEFV)* |  |  |  |  |  |  |  |  | | |  | |  | |  | |  |  | |  | |  | |
| CC | 44 (34.1) | 0.08 | 42 (25.5) | 0.122 | 43 (34.4) | 0.133 | 46 (33.1) | 0.244 | | | 14 (25.0) | | 0.936 | | 11 (21.2) | | 0.505 | 4 (10.3) | | ***0.004†*** | | 181 (26.2) | |
| CA | 61 (47.3) |  | 91 (55.2) |  | 56 (44.8) |  | 61 (43.9) |  | | | 28 (50.0) | |  | | 29 (55.8) | |  | 29 (74.4) | |  | | 328 (26.2) | |
| AA | 24 (18.6) |  | 32 (20.5) |  | 26 (20.8) |  | 32 (23.0) |  | | | 14 (25.0) | |  | | 12 (23.1) | |  | 6 (15.4) | |  | | 182 (26.3) | |
| rs224225  *(MEFV)* |  |  |  |  |  |  |  |  | | |  | |  | |  | |  |  | |  | |  | |
| TT | 47 (33.6) | 0.138 | 45 (25.3) | ***0.022†*** | 47 (36.9) | 0.085 | 54 (33.3) | 0.222 | | | 14 (25.5) | | 0.848 | | 15 (23.4) | | 0.493 | 5 (11.9) | | ***0.003†*** | | 195 (27.0) | |
| TC | 66 (47.1) |  | 102 (57.3) |  | 58 (44.3) |  | 73 (45.1) |  | | | 28 (50.9) | |  | | 35 (54.7) | |  | 31 (73.8) | |  | | 339 (47.0) | |
| CC | 27(19.3) |  | 31 (17.4) |  | 26 (19.8) |  | 35 (21.6) |  | | | 13 (23.6) | |  | | 14 (21.9) | |  | 6 (14.3) | |  | | 188 (26.0) | |
| rs2254441  *(PSTPIP1)* |  |  |  |  |  |  |  |  | | |  | |  | |  | |  |  | |  | |  | |
| GG | 65 (56.5) | 0.467 | 111 (60.7) | 0.852 | 53 (60.2) | 0.911 | 84 (65.1) | 0.79 | | | 23 (67.6) | | 0.824 | | 25 (59.5) | | 0.724 | 23 (43.4) | | ***0.016†*** | | 345 (62.6) | |
| GA | 44 (38.3) |  | 63 (34.4) |  | 31 (35.2) |  | 41 (31.8) |  | | | 10 (29.4) | |  | | 16 (38.1) | |  | 28 (52.8) | |  | | 183 (33.2) | |
| AA | 6 (5.2) |  | 9 (4.9) |  | 4 (4.5) |  | 4 (3.1) |  | | | 1 (2.9) | |  | | 1 (2.4) | |  | 2 (3.8) | |  | | 23 (4.2) | |
| rs8030698  *(PSTPIP1)* |  |  |  |  |  |  |  |  | | |  | |  | |  | |  |  | |  | |  | |
| CC | 67 (54.9) | 0.109 | 118 (62.1) | 0.502 | 58 (58.0) | 0.402 | 89 (64.5) | 0.715 | | | 24 (66.7) | | 0.442 | | 26 (56.5) | | 0.475 | 25 (45.5) | | ***0.005†*** | | 379 (65.0) | |
| CT | 48 (39.3) |  | 66 (34.7) |  | 37 (37.0) |  | 45 (32.6) |  | | | 12 (33.3) | |  | | 17 (37.0) | |  | 29 (52.7) | |  | | 179 (30.7) | |
| TT | 7 (5.7) |  | 6 (3.2) |  | 5 (5.0) |  | 4 (2.9) |  | | | 0 (0.0) | |  | | 3 (6.5) | |  | 1 (1.8) | |  | | 25 (4.3) | |
| rs7173067  *(PSTPIP1)* |  |  |  |  |  |  |  |  | | |  | |  | |  | |  |  | |  | |  | |
| AA | 79 (63.2) | 0.804 | 126 (64.6) | 0.86 | 69 (65.7) | 0.822 | 94 (65.7) | 0.271 | | | 28 (71.8) | | 0.41 | | 30 (58.8) | | 0.326 | 26 (51.0) | | ***0.037†*** | | 394 (66.1) | |
| AG | 40 (32.0) |  | 62 (31.8) |  | 33 (31.4) |  | 47 (32.9) |  | | | 11 (28.2) | |  | | 20 (39.2) | |  | 24 (47.1) | |  | | 178 (29.9) | |
| GG | 6 (4.8) |  | 7 (3.6) |  | 3 (2.9) |  | 2 (1.4) |  | | | 0 (0.0) | |  | | 1 (2.0) | |  | 1 (2.0) | |  | | 24 (4.0) | |
| rs4078354  *(PSTPIP1)* |  |  |  |  |  |  |  |  | | |  | |  | |  | |  |  | |  | |  | |
| CC | 39 (33.6) | ***0.026†*** | 90 (45.9) | 0.945 | 41 (41.4) | 0.153 | 67 (46.5) | 0.949 | | | 21 (56.8) | | 0.29 | | 17 (33.3) | | 0.145 | 13 (23.6) | | ***0.002†*** | | 252 (42.9) | |
| CT | 54 (46.6) |  | 81 (41.3) |  | 51 (51.5) |  | 60 (41.7) |  | | | 11 (29.7) | |  | | 29 (56.9) | |  | 37 (67.3) | |  | | 238 (40.5) | |
| TT | 23 (19.8) |  | 25 (12.8) |  | 7 (7.1) |  | 17 (11.8) |  | 5 (13.5) | | |  | | 5 (9.8) | |  | | 5 (9.1) |  | | 97 (16.5) | |  |

*Persistent oligoarticular juvenile idiopathic arthritis (JIA) affects ≤4 joints; extended oligoarticular JIA affects ≥5 joints after 6 months. Rheumatoid factor (RF)–negative and RF-positive polyarticular JIA affect ≥5 joints within 6 months. All JIA patients were classified according to the criteria of the International League of Associations for Rheumatology (1). SNPs = single-nucleotide polymorphisms.

**†** Statistically significant uncorrected *P* (*P*_uncorr_*)* value versus controls.
